# Characterization and evolution of the *GH3* gene family in *Nymphaea colorata*

**DOI:** 10.3389/fpls.2026.1790058

**Published:** 2026-04-23

**Authors:** Ziyu Li, Zhaohao Zhang, Shinan Li, Jiang Xie, Yuhong Rong, Hongjun Mu

**Affiliations:** Research Center for Tropical Plant Physiology and Ecology, Yunnan Institute of Tropical Crops, Xishuangbanna, Yunnan, China

**Keywords:** evolution, expansion, *GH3*, *Nymphaea colorata*, regulatory network

## Abstract

**Introduction:**

*Gretchen Hagen 3* (*GH3*) genes play important roles in auxin homeostasis, plant development, and stress responses. However, their characterization and evolutionary history in early-diverging angiosperms remain poorly understood.

**Methods:**

Here, we performed a genome-wide identification and comparative evolutionary analysis of the *GH3* gene family in the early-diverging flowering plant *Nymphaea colorata*. Phylogenetic classification, conserved motif and gene structure analyses, promoter cis-element prediction, regulatory network inference, expression profiling, and duplication/selection analyses across 22 plant species were conducted.

**Results:**

A total of 27 *GH3* genes were identified in *N. colorata* and classified into three conserved groups, with 23 *NcGH3* genes assigned to Group 2. Promoter analysis revealed abundant hormone-related and light-responsive cis-elements in *NcGH3* promoters, and regulatory network prediction suggested that Dof transcription factors may act as candidate regulators of NcGH3 genes. Expression analysis showed that most *NcGH3* genes were preferentially expressed in floral organs, including petals, sepals, stamens, juvenile flowers, and carpels. Comparative analysis across 22 plant species indicated that Group 1 represents the most ancestral *GH3* lineage, whereas Groups 2 and 3 emerged later during plant evolution. Group-specific expansion was observed in several species and was mainly associated with whole-genome/segmental duplication and tandem duplication events.

**Conclusion:**

These findings provide a comprehensive overview of the *GH3* gene family in *N. colorata* and offer new insights into the evolutionary diversification and subgroup-specific expansion of *GH3* genes in plants.

## 1 Introduction

Water lilies are aquatic plants with colorful flowers and belong to the earliest-diverging lineages of angiosperms, which are important for studying early evolutionary events within the angiosperms (Zeng et al., 2014). The water lily species *Nymphaea colorata* has a relatively small genome size and blue petals, making it popular in scientific research. The first genome of water lily (*N. colorata*) was released in 2020 (Zhang et al., 2020), providing a foundation for the systematic analysis of gene family evolution and expansion (Khan et al., 2025; Kuang et al., 2025; Liu et al., 2025). And the previous study demonstrated that genes related to immunity and stress responses were significantly expanded in *N. colorata*, including the *Gretchen Hagen 3* (*GH3*) genes, compared with those in *Amborella trichopoda* and some angiosperms (Zhang et al., 2020).

The *GH3* genes encode acyl acid–amido synthetases, which can conjugate excess indole-3-acetic acid (IAA) to amino acids, thereby regulating auxin homeostasis (Wojtaczka et al., 2022). Since the first *GH3* gene was identified as an auxin-responsive gene (Hagen et al., 1984), extensive functional studies have established GH3 proteins as central regulators of hormone-mediated developmental and stress-responsive pathways across land plants. Comparative genomic analyses suggest that *GH3* genes appeared early in plant evolution, and that their subsequent diversification was largely driven by whole-genome and tandem duplications, resulting in lineage-specific expansions (Zhang et al., 2018). In plants, the *GH3* genes were generally grouped into three groups (Group1-3), which differ in gene architecture, expression profiles, and evolutionary history (Wojtaczka et al., 2022; Wang et al., 2024; Guo et al., 2025). However, the current studies on *GH3* genes were mainly focused on model species or major crops, while the evolutionary history of *GH3* genes and their characterization in early-diverging angiosperms remain largely unexplored.

In this study, we systematically identified *GH3* genes in *N. colorata* and performed comparative analyses across 22 representative plant species ranging from algae to angiosperms. We present a comprehensive study on the characteristics of *NcGH3* genes, potential regulatory network, expression profiles and evolution history. Our findings revealed that *GH3* genes in *N. colorata* have experienced group-specific expansion. This study revealed that the characteristics of *GH3* genes in *N. colorata* and provide novel insights into their evolutionary history in plants.

## 2 Materials and methods

### 2.1 Identification of *GH3* genes across plant species

The *GH3* genes in *N. colorata* were identified using BLASTP v2.17.0 and HMM-search v3.4 (Camacho et al., 2009; Eddy, 2011). The *N. colorata* genome assembly and gene annotation were obtained from the published reference (Zhang et al., 2020). First, the annotated GH3 protein sequences from *Arabidopsis thaliana* were used as queries to identify candidate *GH3* genes in *N. colorata* using BLASTP. Then, the HMM profile for the conserved GH3 domain (PF03321) was downloaded from Pfam and used to scan the *N. colorata* protein dataset. All non-redundant candidates were then validated using the NCBI Conserved Domain Database (CDD) to confirm the presence of the GH3 domain (Lu et al., 2020). The validated members were considered as *GH3* genes in *N. colorata* and used for subsequent analyses. Conserved motifs in full-length NcGH3 proteins were analyzed using MEME v5.5.9 (Bailey et al., 2009). To investigate the evolutionary history of *GH3* genes, we applied the same identification pipeline to 22 plant species. We selected them non-randomly to ensure broad evolutionary coverage. These species were chosen to cover all major green plant lineages, including green algae, bryophytes, lycophytes, gymnosperms, basal angiosperms, monocots, and eudicots (**Supplementary Table S1**). For consistency, we used widely accepted, publicly available reference genomes and gene annotations. All genome sequences and annotation files were obtained from NCBI and Phytozome (**Supplementary Table S1**).

### 2.2 Phylogenetic analysis of GH3 proteins

The identified GH3 protein sequences from the 22 plant species were used for phylogenetic analysis. Full-length amino acid sequences were aligned using MAFFT v7.526 with default parameters (Katoh and Standley, 2013). Poorly aligned regions were removed using TrimAl v1.5 with the automated1 option (Capella-Gutiérrez et al., 2009). Maximum-likelihood phylogenetic trees were inferred using IQ-TREE (v3.0.1) based on the trimmed alignments, with the best-fit substitution model selected by ModelFinder. Branch support was assessed using 1,000 ultrafast bootstrap replicates and 1,000 SH-aLRT replicates (Nguyen et al., 2015). Phylogenetic trees were visualized and annotated using iTOL v7 (Letunic and Bork, 2024). Based on phylogenetic relationships and group information for *GH3* genes in *A. thaliana*, we classified *GH3* genes into three conserved subgroups (Group 1–3).

### 2.3 Chromosomal localization and synteny analysis

We retrieved the chromosomal position of *NcGH3s* directly from the genome annotation files. To analyze collinearity, we performed a genome-wide synteny analysis in *N. colorata*. Protein sequences were compared against each other in an all-against-all approach, and collinear blocks were identified using MCScanX v1.0.0 with default settings (Wang et al., 2012). Finally, we visualized the chromosomal locations of GH3 genes and their syntenic relationships using TBtools v2.388 (Chen et al., 2023).

### 2.4 Cis-acting element analysis and transcriptional regulatory network construction

To investigate the transcriptional regulation of NcGH3s, we extracted 2,000 bp of upstream promoter sequences and scanned them for cis-acting elements. We identified these elements using the PlantCARE database (Lescot et al., 2002). Next, we predicted potential transcription factor (TF) binding sites in NcGH3 promoters. Due to the limited availability of experimentally validated TF–DNA interactions for *N. colorata*, we used *A. thaliana* TF binding motifs from PlantTFDB as references (Jin et al., 2017). Then, we used FIMO (MEME Suite v5.5.9) to search for these motifs in 2,000 bp promoter regions, using a threshold of 1.0E-5 (Grant et al., 2011).

### 2.5 miRNA-mediated regulatory network analysis

To evaluate post-transcriptional regulation of NcGH3s, we predicted the miRNA-mediated interactions for NcGH3s. Mature miRNA sequences from *N. colorata* were retrieved from ANAgdb (**Supplementary Table S2**) (Guo et al., 2024). Potential miRNA–target pairs were identified using psRNATarget (Dai et al., 2018). Candidate interactions were filtered by expectation score (score > 4) to retain only high-confidence regulatory relationships.

### 2.6 Expression profiling and co-expression analysis of *NcGH3s*

Transcriptomic data for *N. colorata* were obtained from the published genome study (**Supplementary Table S3**) (Zhang et al., 2020). Gene expression levels were quantified as transcripts per million (TPM). To identify genes co-expressed with *NcGH3s*, pairwise correlation analysis was performed between *NcGH3s* and all expressed genes across samples. Spearman’s rank correlation coefficient was used to assess expression correlation. Genes showing a strong correlation with *NcGH3s*, defined as an absolute correlation coefficient (|r|) greater than 0.6 and a significance level of *P* < 0.05, were considered co-expressed genes. Gene Ontology (GO) enrichment analysis was subsequently performed on the identified co-expressed genes using the R package clusterProfiler (Yu et al., 2012).

### 2.7 Gene duplication and subgroup expansion analysis

To investigate the mechanisms underlying *GH3* gene expansion, duplication modes of *GH3* genes were identified in representative plant species using MCScanX (Wang et al., 2012). Genome-wide duplication events were assigned to whole-genome/segmental duplication (WGD/SD), tandem duplication (TD), proximal duplication, or dispersed duplication according to gene positions and inferred syntenic relationships. To assess subgroup-specific expansion, we counted the number of GH3 genes in each subgroup for all surveyed plant genomes. We then measured expansion as the fold change in subgroup copy number for each species relative to the mean subgroup copy number across the 22 species.

### 2.8 Evolutionary rate and selection pressure analysis

To assess evolutionary constraints on duplicated *GH3* genes, we calculated synonymous (Ks) and nonsynonymous (Ka) substitution rates for each gene pair using KaKs Calculator v3.0 (Zhang, 2022). We analyzed gene pairs from WGD/SD and TD. Gene pairs with unreliable estimates, such as zero or undefined Ks values, were excluded from further analysis.

## 3 Results

### 3.1 Identification and chromosomal organization of *GH3* genes in *N. colorata*

**Figure 1:**
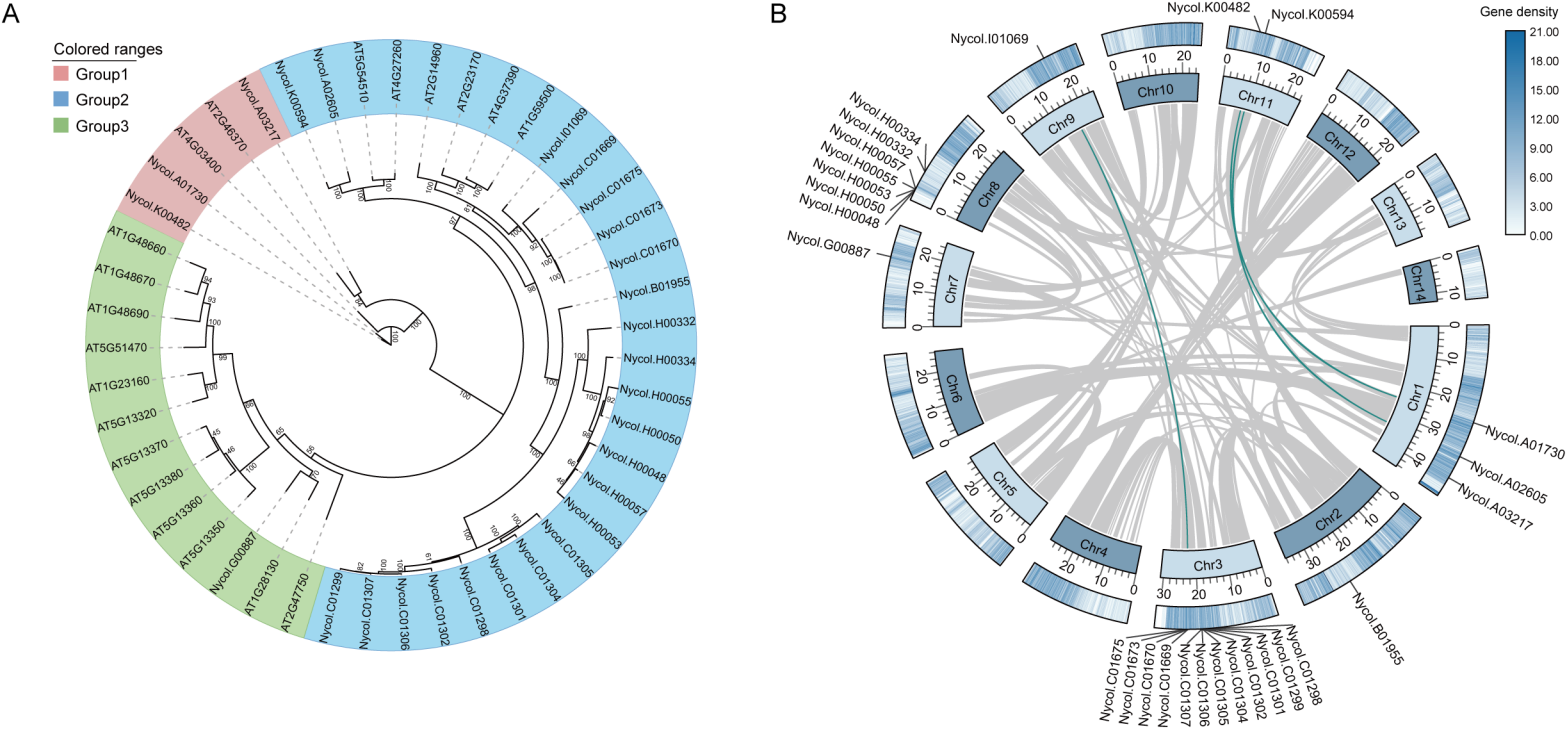
Phylogenetic relationships and chromosomal organization of GH3 genes in *Nymphaea colorata*. **(A)** Maximum-likelihood phylogenetic tree of GH3 proteins from *Nymphaea colorata*. Branch colors indicate GH3 groups (Group 1–3). Bootstrap support values are shown at the nodes, with dot size proportional to support strength. **(B)** Circos representation of the chromosomal distribution and syntenic relationships of GH3 genes in *N. colorata*. Chromosomes are displayed in a circular layout, with gene density indicated by the outer histogram track. Gray links denote genome-wide syntenic blocks, whereas highlighted links indicate collinear relationships among GH3 loci. Gene names are labeled on the corresponding chromosomal positions.

A total of 27 *GH3* genes were identified in *N. colorata* (**Supplementary Table S4**). Among them, although two genes (*Nycol.C01669* and *Nycol.H00053*) appeared to contain an incomplete GH3 domain, they were considered as putative *GH3* genes to provide a comprehensive overview of the *GH3* genes in *N. colorata*. An ML phylogenetic tree was constructed using the 27 NcGH3s and 20 AtGH3s protein sequences (**Figure 1A**), and then classified these GH3s into three groups (Group 1–3). Notably, the member of the three groups varies markedly, with Group 2 being the largest in *N. colorata* (23/27), compared to Group 3 in *A. thaliana* (12/20). This group-specific clustering was well supported by bootstrap values, indicating the different evolutionary strategy of GH3 between *N. colorata* and *A. thaliana*. In addition, *NcGH3s* were unevenly distributed across the chromosomes, with chr3 (12) and chr8 (7) containing the most *GH3* genes, indicating a non-random chromosomal organization (**Figure 1B**). There were three *NcGH3* gene pairs located within syntenic blocks (**Figure 1B**), indicating a limited contribution of large-scale duplication events to *GH3* gene expansion in *N. colorata*.

### 3.2 Conserved motifs and gene structure of *NcGH3s*

**Figure 2:**
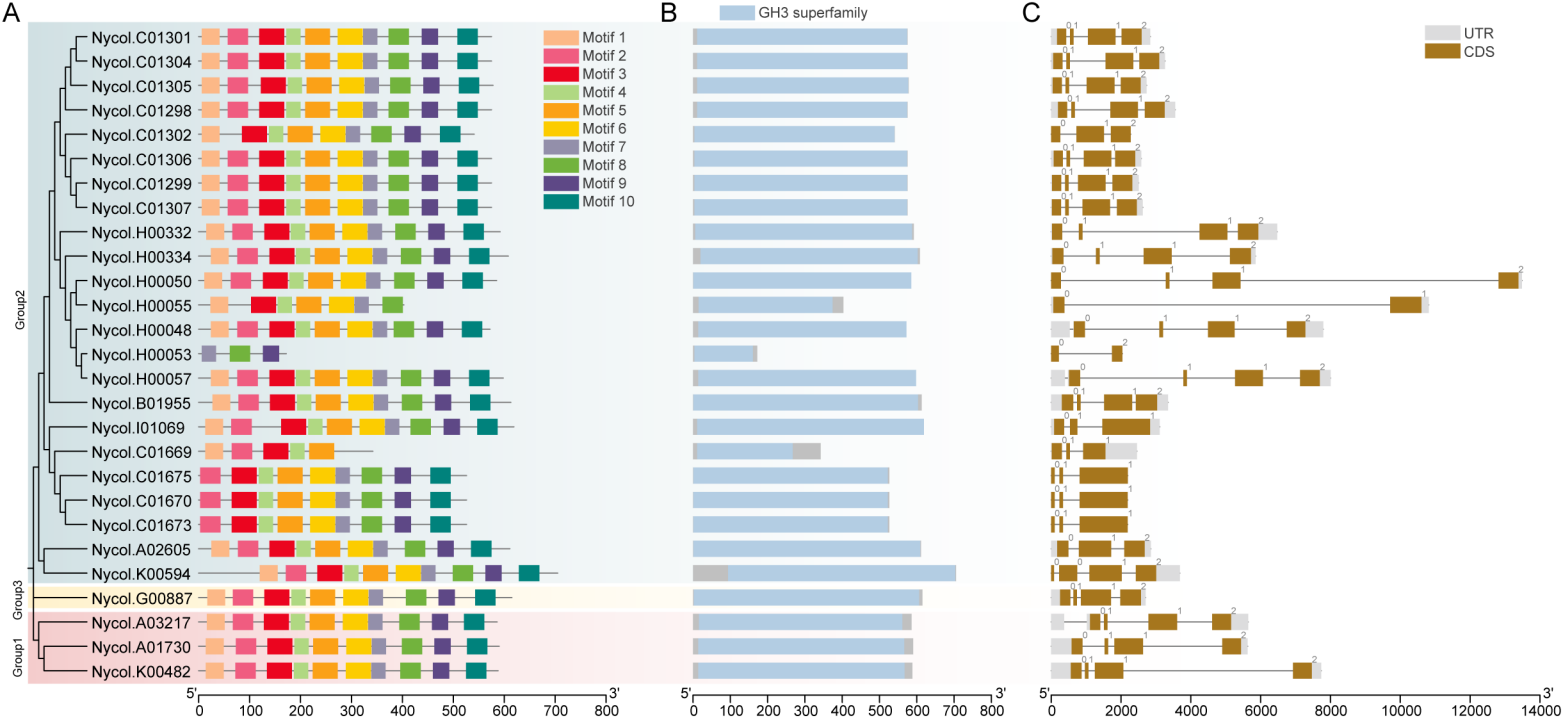
Conserved motifs and gene structures of GH3 genes in *N. colorata*. **(A)** Distribution of conserved motifs among NcGH3 proteins. Ten conserved motifs were identified and are shown as colored boxes. Proteins are ordered according to the phylogenetic relationships shown on the left, and group classifications (Group 1–3) are indicated by background shading. **(B)** Domain composition of NcGH3 proteins. The presence of the GH3 superfamily domain is shown for each protein, illustrating the overall conservation of the core GH3 domain across NcGH3 members. **(C)** Exon–intron structures of *NcGH3s*. Exons are shown as filled boxes and introns as connecting lines, with untranslated regions (UTRs) indicated in gray. Gene structures are aligned according to the corresponding phylogenetic order.

The structural characteristics of *NcGH3s* were examined by identifying 10 conserved motifs through the MEME database. Most of the NcGH3 members shared common motif compositions and arrangement, indicating strong structural conservation of the NcGH3s (**Figure 2A**). For Nycol.H0053 and Nycol.C01669, the motif composition of their current sequences is also similar to that of other NcGH3s. Analysis of protein domain composition confirmed that all NcGH3 proteins contained a complete GH3 superfamily domain except Nycol.H0053 and Nycol.C01669 (**Figure 2B**). Gene structure analysis revealed that closely related *NcGH3s* shared similar exon–intron organization (**Figure 2C**). Despite the presence of a conserved motif in Group 2 members, the gene structure patterns exhibited significant variation, suggesting a potential for functional differentiation within Group 2 members of NcGH3.

### 3.3 Cis-acting elements and transcriptional regulatory networks of *NcGH3s*

**Figure 3:**
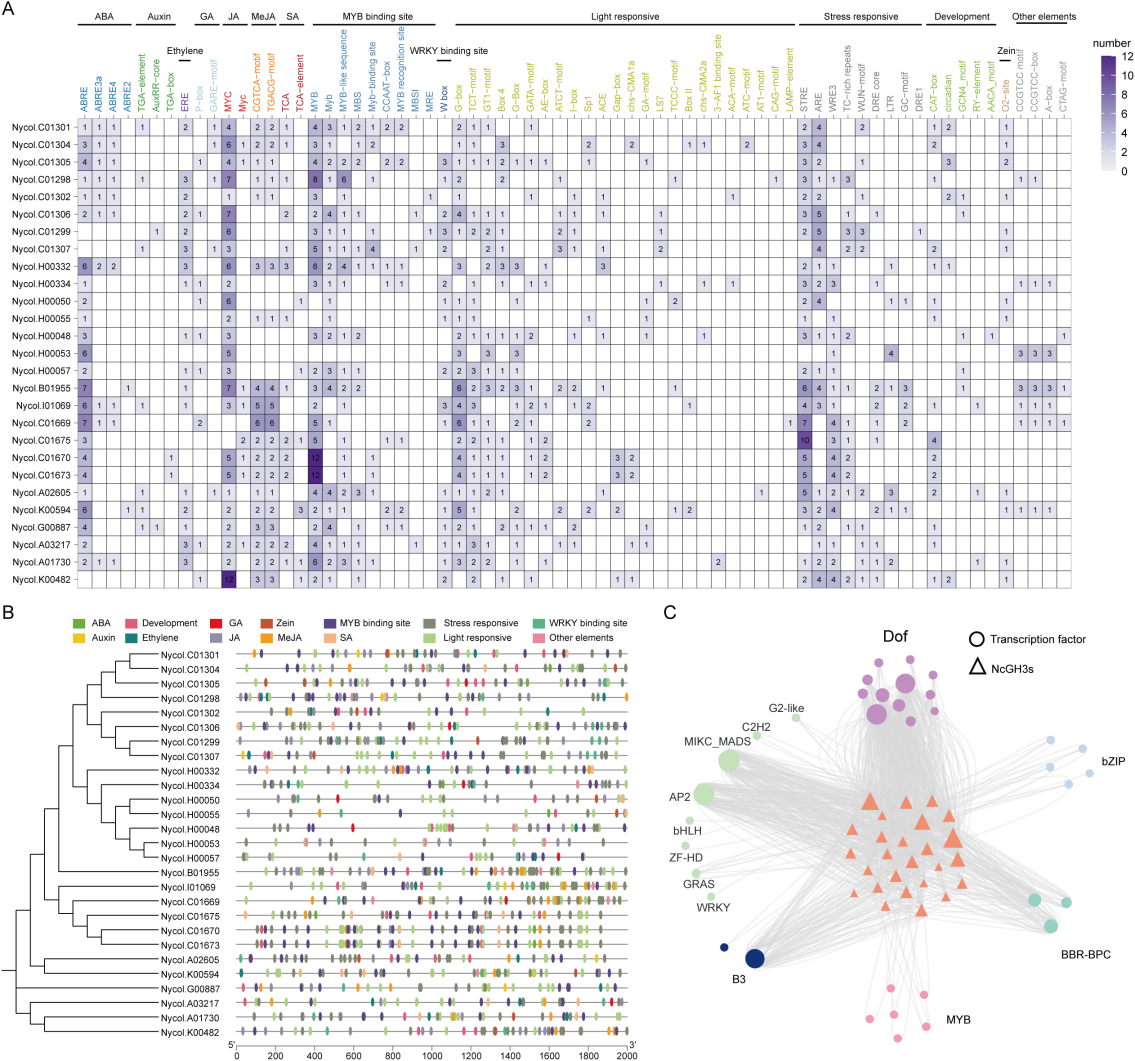
Cis-regulatory elements and predicted transcriptional regulatory network of *NcGH3s*. **(A)** Heatmap showing the abundance of cis-regulatory elements identified in the promoter regions of *NcGH3s*. Promoter sequences of 2,000 bp upstream of the transcription start site were analyzed, and cis-elements were grouped according to hormone responsiveness, light responsiveness, stress responsiveness, developmental regulation, and other functional categories. Numbers within cells indicate the occurrence of each cis-element. **(B)** Distribution of cis-regulatory elements along the promoter regions of *NcGH3s*. Different colors represent distinct cis-element categories, and genes are arranged according to their phylogenetic relationships. **(C)** Predicted transcriptional regulatory network of *NcGH3s*. Nodes represent transcription factors (circles) and *NcGH3s* (triangles), with edges indicating predicted regulatory interactions. Transcription factors are colored according to their family classification.

The cis-acting elements in the promoter region of *NcGH3* genes were explored, which play important roles in regulating gene expression. In total, 1,282 cis-acting elements were identified in the promoters of *NcGH3s* (**Figure 3A**). The hormone-related elements were widely distributed in the promoter of *NcGH3* genes, including those responsive to auxin (11), abscisic acid (102), gibberellin (15), jasmonic acid (115), salicylic acid (28), ethylene (32), zein (13) and MeJA (90). In addition to hormone-responsive elements, a large number of light-responsive and stress-related elements were detected, indicating a potential role of *NcGH3s* in environmental signal integration. The number and composition of elements differed markedly among genes, indicating the expression divergence in *NcGH3* genes. Promoters of phylogenetically closely related *NcGH3s* tended to exhibit more similar element composition (**Figures 3A, B**). To further explore the transcriptional regulation on *NcGH3* genes, a transcriptional regulatory network was constructed to identify potential TF–GH3 interactions (**Figure 3C**). This predicted network comprises 277 regulatory relationships involving 36 TFs and 27 NcGH3s. Notably, there were 12 Dof TFs that exhibited 148 potential regulatory relationships with 25 *NcGH3* genes (**Figure 3C**). In addition, the six predicted MYB TFs further supported the widely distributed MYB binding sites (**Figure 3A**), indicating the accuracy of our predicted network.

### 3.4 miRNA-mediated post-transcriptional regulatory network of *NcGH3s*

**Figure 4:**
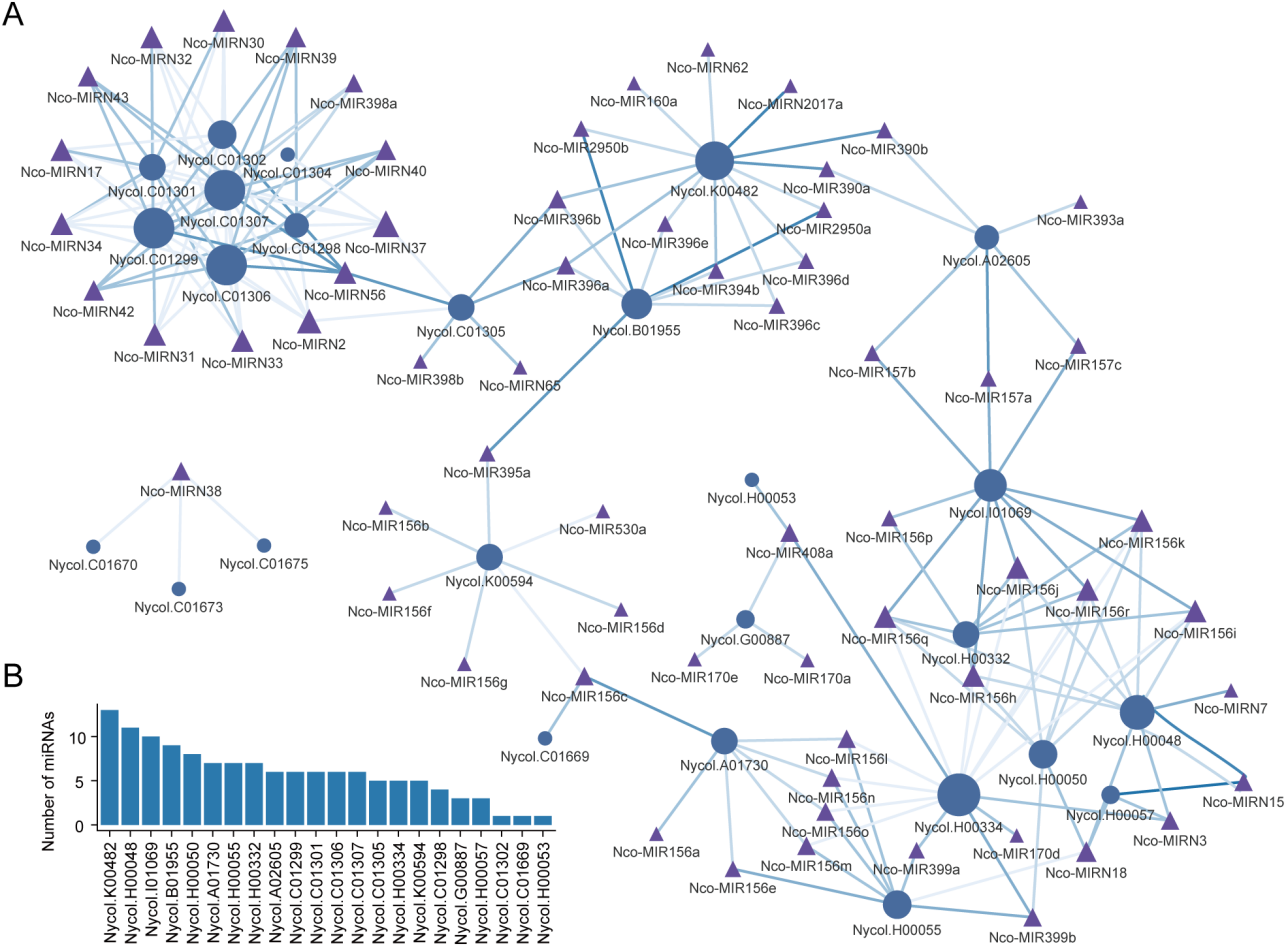
Predicted miRNA–mediated regulatory network of NcGH3s. **(A)** Predicted miRNA–NcGH3 regulatory network in *N. colorata*. Circular nodes represent *NcGH3s*, whereas triangular nodes denote miRNAs. Edges indicate predicted regulatory interactions between miRNAs and their target *NcGH3s*. Node size reflects the number of interactions, highlighting *NcGH3s* targeted by multiple miRNAs. **(B)** Number of miRNAs for each NcGH3 gene. Bars indicate the total number of miRNAs associated with individual *NcGH3s*, illustrating variation in miRNA-mediated regulatory complexity among NcGH3 family members.

Plant miRNAs were generally reported to regulate gene expression, and miRNA involvement in auxin-associated pathways has been reported (Mallory et al., 2005). To assess whether miRNAs are involved in the post-transcriptional regulation of *NcGH3* genes, we used psRNATarget to predict the putative miRNA–GH3 interaction network in *N. colorata* (**Figure 4A**). The predicted regulatory network linked 60 miRNAs to 22 *NcGH3* genes. Notably, 65% of the miRNAs were predicted to target at least two *NcGH3* members, and 19 *NcGH3*s were associated with at least two miRNAs, suggesting extensive interactions between miRNAs and *NcGH3* genes (**Figure 4B**).

### 3.5 Tissue-specific expression pattern and co-expression analysis of *NcGH3s*

**Figure 5:**
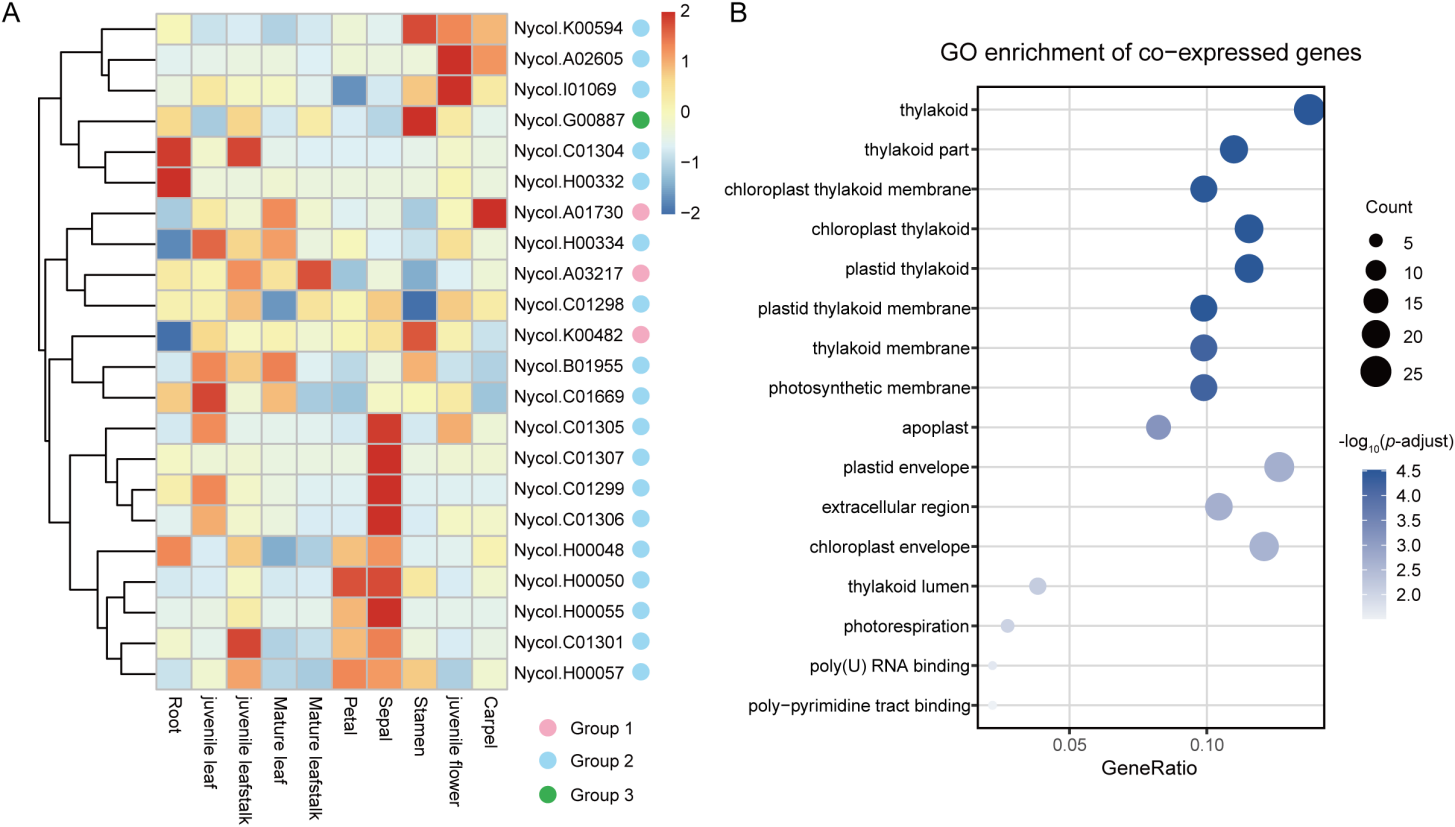
Tissue-specific expression patterns of NcGH3s and functional enrichment of co-expressed genes. **(A)** Heatmap showing the expression profiles of *NcGH3s* across different tissues of *N. colorata*. Expression levels were scaled by gene to highlight relative expression differences among tissues. **(B)** Gene Ontology (GO) enrichment analysis of genes co-expressed with *NcGH3s*. Enriched GO terms are shown with gene ratio on the x-axis and GO categories on the y-axis. Dot size indicates the number of genes associated with each term, and color represents the significance level.

To investigate the expression pattern, we analyzed the expression levels of *NcGH3* genes across ten tissue types in *N. colorata*, and 22 *NcGH3* genes were detected to be expressed in at least one tissue (**Figure 5A**). The three genes in Group 1 exhibited divergent expression patterns, with preferential expression in carpel, stamen, and mature leafstalk, respectively. Similarly, genes in Group 2 exhibited several distinct expression patterns, although some members showed similar expression profiles. For example, *Nycol.C01305*, *Nycol.C01306*, *Nycol.C01307*, and *Nycol.C01299* were predominantly expressed in the sepal, whereas *Nycol.C01304* and *Nycol.H0032* were preferentially expressed in the root. Meanwhile, the sole member of Group 3 was preferentially expressed in the stamen. To investigate the broader functional role of *NcGH3s*, co-expression analysis was performed using Spearman’s rank correlation. A total of 337 genes showed strong expression correlations with 15 *NcGH3s*, including 152 positive co-expressed genes and 185 negative co-expressed genes (**Supplementary Table S5**). Notably, *Nycol.K00594* had the largest number of co-expressed genes (174), which showed predominant expression in flower organs (stamen, juvenile flower, and carpel). Gene Ontology (GO) enrichment analysis showed that these co-expressed genes were related to photosynthesis-associated processes, including thylakoid organization, photosynthetic membrane components, and chloroplast-associated structures (**Figure 5B**).

### 3.6 Evolutionary expansion of *GH3* genes across 22 plant species

**Figure 6:**
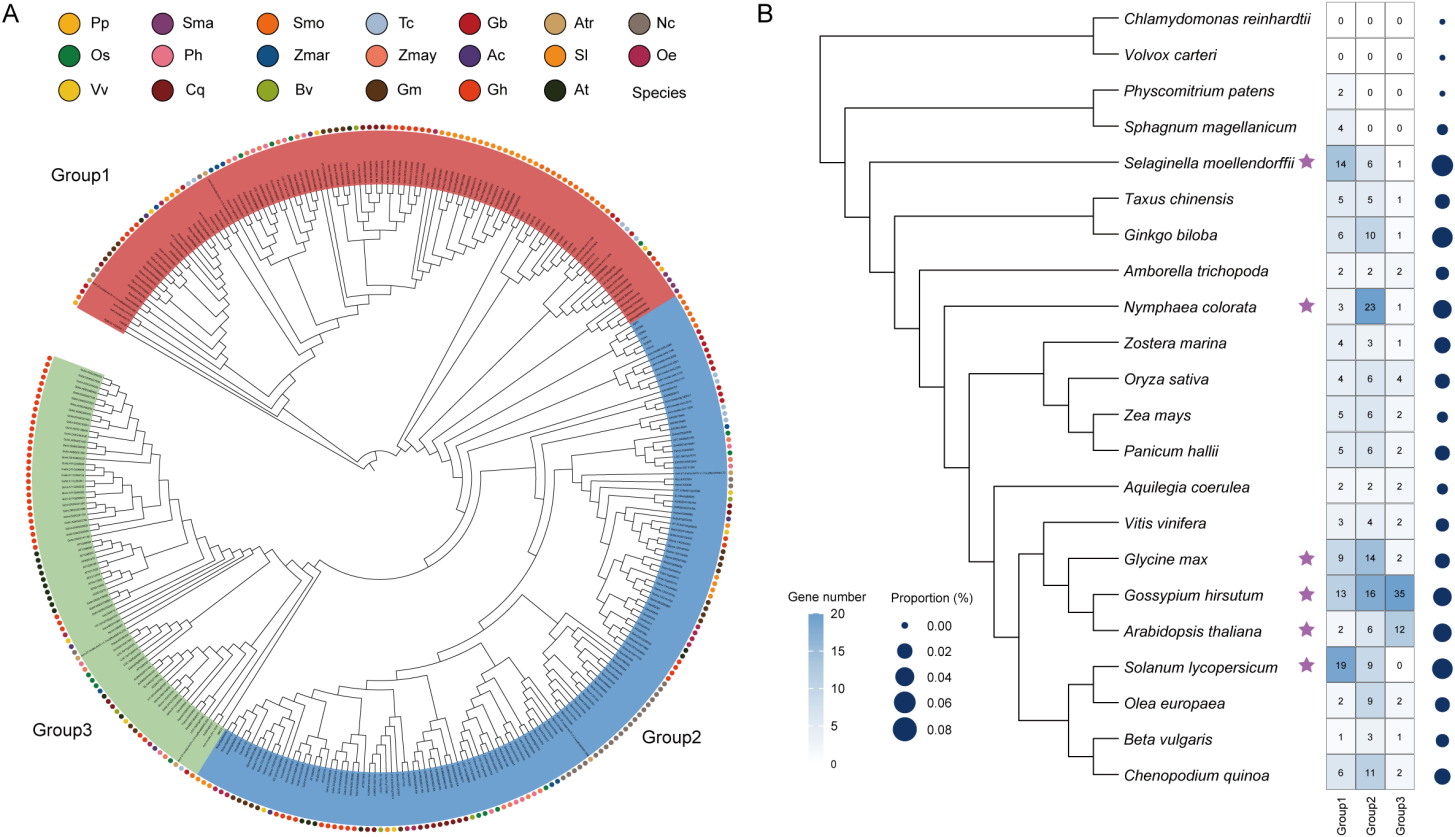
Evolutionary distribution and phylogenetic relationships of GH3 genes across representative plant species. **(A)** Maximum-likelihood phylogenetic tree of 325 GH3 proteins from the 22 representative plant species. The dots surrounding the tree represent species. **(B)** Distribution of *GH3* genes across 22 plant species. The phylogenetic relationships of species are shown on the left. Heatmap indicates the number of *GH3* genes in each subgroup (Group 1–3), while dot size represents the proportion of *GH3* genes relative to the total number of annotated genes in each genome.

To investigate the evolutionary history of *GH3* genes, we performed a comparative analysis of the *GH3* genes across 22 plant species covering major green plant lineages, including algae, bryophytes, lycophytes, gymnosperms, and angiosperms (**Figure 6A**). A total of 325 GH3 genes were identified, ranging from two members in *Physcomitrium patens* to 64 members in *Gossypium hirsutum* (**Supplementary Table S6**). Plant *GH3* genes were absent from the two chlorophyte algae examined (*Chlamydomonas reinhardtii* and *Volvox carteri*), whereas GH3 members were readily detected in land plants (**Figure 6B**). Group 1 represents the ancestral type, originating in bryophytes and subsequently evolving into Groups 2 and 3 in ferns. Notably, we observed a significant imbalance in the three groups of *GH3* genes in most species (**Figure 6B**). For example, 66.7% of the GH3 genes in *Selaginella moellendorffii* were classified into Group 1, 85.2% of those in *N. colorata* into Group 2, and 54.7% of those in *G. hirsutum* into Group 3. And six species displayed significant expansion in a specific group (**Supplementary Figure S1**), including *S. moellendorffii* (Group 1), *N. colorata* (Group 2), *Glycine max* (Group 2), *G. hirsutum* (Group 1/2/3), *A. thaliana* (Group 2), and *Solanum lycopersicum* (Group 1).

### 3.7 Duplication patterns and Ka/Ks analysis in expanded *GH3* genes

**Figure 7:**
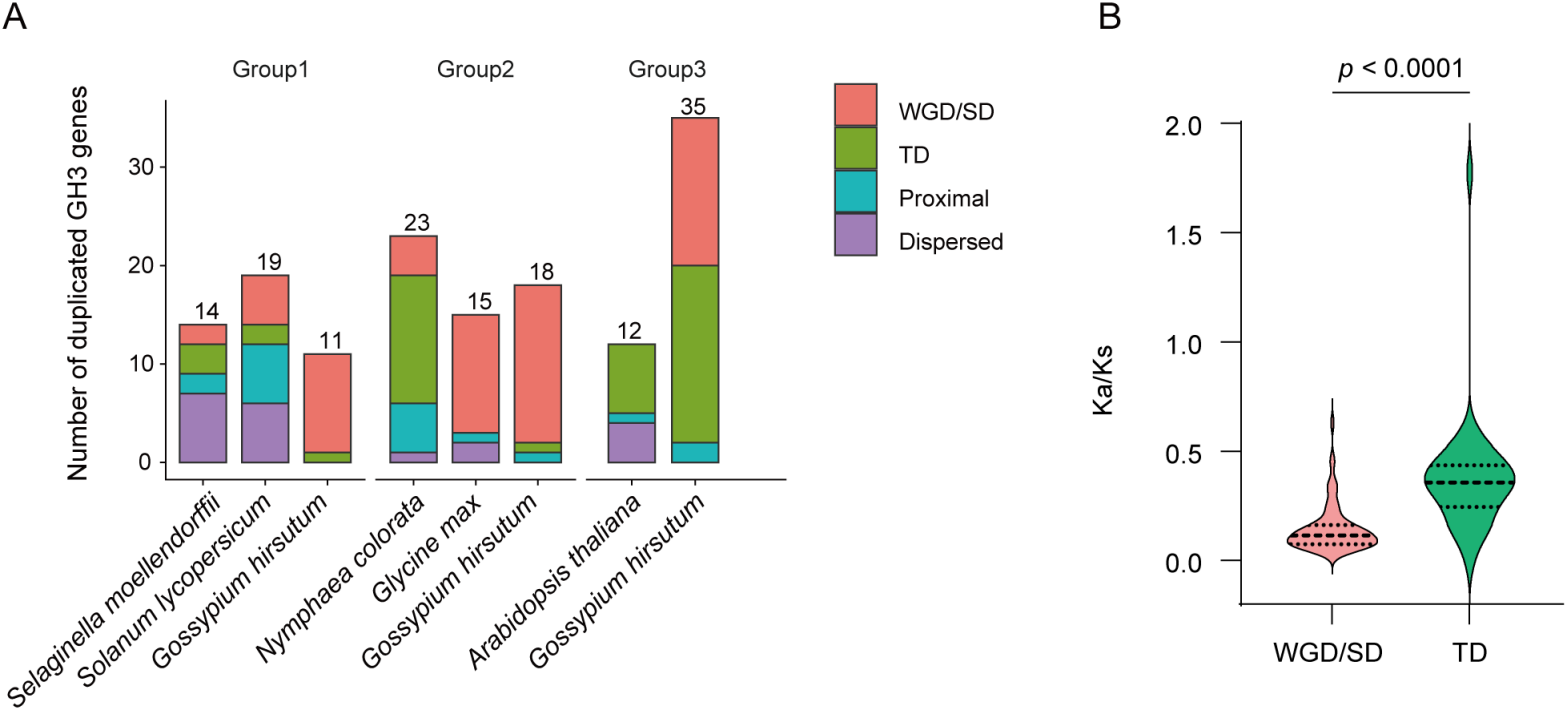
Duplication patterns and selective constraints of expanded GH3 subgroups. **(A)** Duplication types of *GH3* genes in significantly expanded groups. The number of duplicated *GH3* genes is shown for each subgroup, with colors indicating different duplication modes, including whole-genome or segmental duplication (WGD/SD), tandem duplication (TD), proximal duplication, and dispersed duplication. Only species exhibiting notable subgroup expansion are shown. **(B)** Distribution of Ka/Ks ratios for *GH3* gene pairs derived from WGD/SD and TD events. Horizontal lines indicate median Ka/Ks values for each category. Statistical significance between categories was evaluated using the Wilcoxon rank-sum test.

To explore the expansion mechanisms of *GH3* genes, we investigated the duplication pattern of *GH3* genes in six species with significant group expansion (**Figure 7A**). Distinct duplication patterns were observed in different groups (**Supplementary Table S7**). The duplication pattern exhibited species-specificity. For example, whole-genome or segmental duplication (WGD/SD) was the main driving force of the *GH3* genes’ expansion in *G. hirsutum*, whereas tandem duplication (TD) contributed substantially to the expansion of Group 2 members in *N. colorata*. For a further understanding of GH3 evolutionary relationships, we calculated the ratio of Ka/Ks for each WGD/SD- and TD-derived gene pairs (**Supplementary Table S8**). Overall, the Ka/Ks ratios of *GH3* gene pairs were less than 1, indicating that *GH3* genes were subject to purifying selection. However, the Ka/Ks value of *Gohir.D04G027100* and *Gohir.D04G027200* gene pair is 1.775, suggesting a positive selection pressure. Furthermore, the Ka/Ks ratio for TD-driven gene pairs was significantly higher than that for WGD-driven gene pairs, indicating relatively relaxed constraints and faster sequence divergence following TD (**Figure 7B**; **Supplementary Figure S2**).

## 4 Discussion

*N. colorata* is a member of Nymphaeales, an early-diverging angiosperm lineage that offers a valuable resource for studying the evolutionary history of plant gene families. A previous comparative analysis reported that *GH3* genes exhibited significant expansion in *N. colorata* (Zhang et al., 2020). A total of 27 *NcGH3* genes were consistently identified, indicating a higher gene count compared to the *GH3* genes in species *A. tricopoda* and *A. thaliana*. A remarkably high proportion (85.2%) of Group 2 members was identified in *N. colorata*, and these genes generally showed preferential expression across different floral tissues, including juvenile flowers, stamens, and sepals (**Figure 5A**). To further understand the evolutionary history, we identified 325 *GH3* genes in 22 plant species covering chlorophyte algae, bryophytes, lycophytes, gymnosperms and angiosperms. Consistent with a previous study (Zhang et al., 2018), *GH3* genes, absent in chlorophyte algae, emerged in the earliest land plants (*P. patens* and *S. magellanicum*) and rapidly expanded in angiosperms. This origin pattern generally occurred in plant gene families, such as Auxin Response Factor (ARF) genes (Finet et al., 2013). Among plant *GH3* genes, Group 1 represents the ancestral lineage associated with JA and may function in the environmental adaptation of early land plants (Zhang et al., 2018). The *GH3* genes in Group 2 and 3 first emerged in ferns and subsequently underwent rapid expansion in angiosperms. The members of Group 2 were related to IAA (Luo et al., 2023), while those in Group 3 were involved in the response to biotic and abiotic stress (Okrent and Wildermuth, 2011). These functional differences underscore the evolutionary diversification of the GH3 family and point to its potential importance in coordinating hormone homeostasis and stress adaptation during plant evolution. Furthermore, the group-specific expansion was observed in several species, such as Group 1 in *S. moellendorffii*, Group 2 in *N. colorata*, and Group 3 in *G. hirsutum*. These group-specific expansions were mainly driven by WGD/SD or TD events, with the relatively relaxed selective constraints and faster sequence divergence in TD. Taken together, the evolutionary analysis of *GH3* genes in *N. colorata* provides new insights into subgroup-specific expansion during plant evolution. 

## Data Availability

The original contributions presented in the study are included in the article/**Supplementary Material**. Further inquiries can be directed to the corresponding authors.
