# Supplementary material for: Characterization and evolution of the GH3 gene family in Nymphaea colorata
Source: Front Plant Sci. 2026 Apr 23;17:1790058. doi: 10.3389/fpls.2026.1790058 (PMC13149400; doi:10.3389/fpls.2026.1790058)
Supplement: Supplementary file 1 [file SupplementaryFile1.docx]

**Supplementary Figures**

**
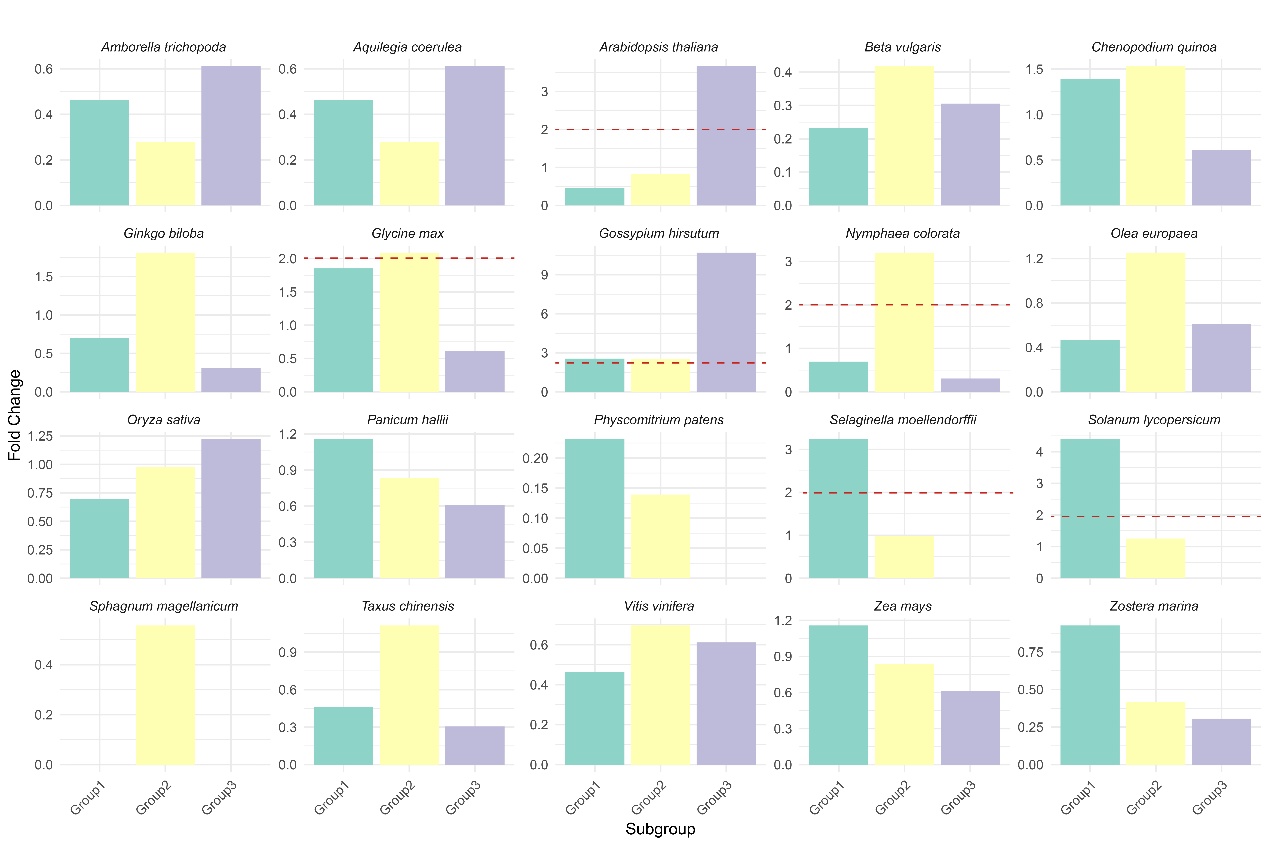
**

**Supplementary Figure S1. Subgroup-specific expansion of GH3 genes across 20 plant species.** Fold change of GH3 gene numbers in each subgroup across representative plant species. For each subgroup, the fold change was calculated by normalizing the GH3 gene copy number in a given species to the mean copy number of that subgroup across all analysed species. Bars indicate subgroup-specific expansion or contraction relative to the overall mean. Each panel represents one species, with colours denoting different GH3 subgroups (Group 1–3). For a subgroup to be considered significantly expanded, the fold change must exceed two.

**
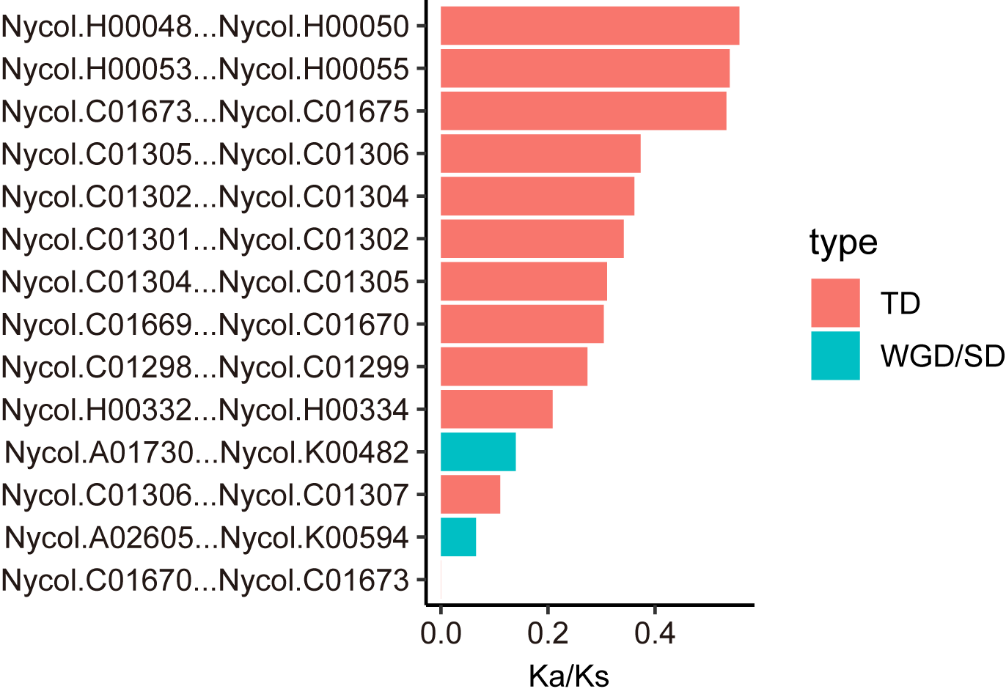
**

**Supplementary Figure S2. Ka/Ks analysis of duplicated GH3 gene pairs within *Nymphaea colorata*.** Ka/Ks ratios of duplicated GH3 gene pairs identified within *N. colorata*. Each bar represents a duplicated gene pair, with colours indicating the corresponding duplication mode, including tandem duplication (TD) and whole-genome or segmental duplication (WGD/SD). Gene pairs are ordered by their Ka/Ks values. Most duplicated gene pairs exhibit Ka/Ks ratios below 1.
